# The Auto-Regulation of ATL2 E3 Ubiquitin Ligase Plays an Important Role in the Immune Response against *Alternaria brassicicola* in *Arabidopsis thaliana*

**DOI:** 10.3390/ijms25042388

**Published:** 2024-02-17

**Authors:** Daewon Kim, Su Jeong Jeon, Jeum Kyu Hong, Min Gab Kim, Sang Hee Kim, Ulhas S. Kadam, Woe-Yeon Kim, Woo Sik Chung, Gary Stacey, Jong Chan Hong

**Affiliations:** 1 Division of Life Science and Division of Applied Life Science (BK21 Four), Plant Molecular Biology and Biotechnology Research Center (PMBBRC), Gyeongsang National University, 501 Jinju-daero, Jinju 52828, Republic of Korea; kimdaew@missouri.edu (D.K.); sujeongjeon83@gmail.com (S.J.J.); sangheekim@gnu.ac.kr (S.H.K.); ukadam@gnu.ac.kr (U.S.K.); 2 Division of Plant Science & Technology, C.S. Bond Life Sciences Center, University of Missouri, Columbia, MO 65211, USA; staceyg@missouri.edu; 3 Laboratory of Horticultural Crop Protection, Division of Horticultural Science, Gyeongsang National University, 33 Dongjin-ro, Jinju 52725, Republic of Korea; jkhong@gnu.ac.kr; 4 Agri-Food Bio Convergence Institute, Gyeongsang National University, 33 Dongjin-ro, Jinju 52725, Republic of Korea; 5 College of Pharmacy and Research Institute of Pharmaceutical Science, Gyeongsang National University, 501 Jinju-daero, Jinju 52828, Republic of Korea; mgk1284@gnu.ac.kr; 6 Division of Applied Life Science (BK21 Four), Plant Biological Rhythm Research Center (PBRRC), Plant Molecular Biology and Biotechnology Research Center (PMBBRC), Gyeongsang National University, 501 Jinju-daero, Jinju 52828, Republic of Korea

**Keywords:** chitin, fungal pathogen, defense response, ubiquitin/26S proteasome, ATL2, RING-H2-type E3 ligase, plasma membrane, *Arabidopsis thaliana*

## Abstract

The ubiquitin/26S proteasome system is a crucial regulatory mechanism that governs various cellular processes in plants, including signal transduction, transcriptional regulation, and responses to biotic and abiotic stressors. Our study shows that the RING-H2-type E3 ubiquitin ligase, *Arabidopsis Tóxicos en Levadura 2* (*ATL2*), is involved in response to fungal pathogen infection. Under normal growth conditions, the expression of the *ATL2* gene is low, but it is rapidly and significantly induced by exogenous chitin. Additionally, ATL2 protein stability is markedly increased via chitin treatment, and its degradation is prolonged when 26S proteasomal function is inhibited. We found that an *atl2* null mutant exhibited higher susceptibility to *Alternaria brassicicola*, while plants overexpressing *ATL2* displayed increased resistance. We also observed that the hyphae of *A. brassicicola* were strongly stained with trypan blue staining, and the expression of *A. brassicicola Cutinase A* (*AbCutA*) was dramatically increased in *atl2*. In contrast, the hyphae were weakly stained, and *AbCutA* expression was significantly reduced in *ATL2*-overexpressing plants. Using bioinformatics, live-cell confocal imaging, and cell fractionation analysis, we revealed that ATL2 is localized to the plasma membrane. Further, it is demonstrated that the ATL2 protein possesses E3 ubiquitin ligase activity and found that cysteine 138 residue is critical for its function. Moreover, ATL2 is necessary to successfully defend against the *A. brassicicola* fungal pathogen. Altogether, our data suggest that ATL2 is a plasma membrane-integrated protein with RING-H2-type E3 ubiquitin ligase activity and is essential for the defense response against fungal pathogens in *Arabidopsis*.

## 1. Introduction

Post-translational ubiquitination of proteins is a crucial regulatory process in eukaryotes, exerting a profound impact on cellular signaling and various biological processes [1]. This modification has the potential to significantly alter various characteristics of proteins, including their subcellular localization, activity, and interaction capabilities. Alternatively, depending on the specific configuration of the ubiquitin chain, it can result in the degradation of target proteins within the 26S proteasome [2,3,4]. Ubiquitin (Ub) is a small polypeptide that consists of 76 highly conserved amino acids capable of being covalently attached to lysine (K) residues on the target substrate protein [5,6]. This attachment is facilitated by a sequential cascade involving three key enzymes: ubiquitin-activating enzyme (E1), ubiquitin-conjugating enzyme (E2), and ubiquitin ligase (E3) [2,7]. The initial step in this cascade involves the ATP-dependent formation of a thioester-linked ubiquitin by the E1 Ub-activating enzyme, followed by the transfer of the thioester-linked ubiquitin domain to a cysteinyl residue on the E2 Ub-conjugating enzyme [2]. Ultimately, an E3 Ub ligase coordinates the transfer of ubiquitin to a lysyl group on the target protein [8,9,10].

There are two subfamilies of E3 ubiquitin ligases, which are distinguished by the presence of either a RING (Really Interesting New Gene)/U box or HECT (Homologous to the E6-AP Carboxyl Terminus) domain, each with distinct domains and mechanisms of action [11]. The RING domain contains essential Cys and His conserved key residues (Cys-X_2_-Cys-X_9-39_-Cys-X_1-3_-His-X_2-3_-Cys/His-X_2_-Cys-X_4-48_-Cys-X_2_-Cys) that form an active site for the E2-ubiquitin intermediate, functioning as a zinc-binding domain. Canonical RING fingers are further classified into two subdomains, C_3_H_2_C_3_ (RING-H2)-type or C_3_HC_4_-type (RING-CH), depending on the fifth conserved amino acid [12,13,14]. The *Arabidopsis* genome contains almost 1300 genes, encoding putative E3 ubiquitin ligases, of which 400 members are of the RING-finger type [4,15]. RING-type E3 ubiquitin ligases are involved in response to environmental stimuli, such as tolerance mechanisms against high salinity, dehydration, osmotic, and freezing stress, as well as in pathogen defense responses [10,16,17,18]. The SKP CDC53p/CUL1 F-box (SCF) complex is one of the most well-characterized RING-type E3 ubiquitin ligases in plant–pathogen interactions [7,19].

The *Arabidopsis* ATL (*Arabidopsis Tóxicos en Levadura*) subfamily contains 91 members that exhibit a highly conserved RING zinc finger domain, which suggests their role as E3 ubiquitin ligase proteins [20]. These proteins contain a transmembrane domain, a condensed basic region, and a conserved region of 16 amino acids, followed by the RING-H2 zinc finger domain [21]. The ATL E3 ubiquitin ligase protein plays a role in various plant defense mechanisms, encompassing not only plant growth and development but also a wide range of abiotic and biotic stresses [22,23,24,25]. Several members of the *ATL* gene family are activated by various elicitors in pathogen defense pathways [21,26]. Elicitors increase the expression of LeATL6 from tomato and may play a role in fungal elicitor-induced defense responses through a jasmonic acid (JA)-dependent signaling pathway [27]. Additionally, *elicitor-responsive 5* (*EL5*), a homolog of the rice *ATL* gene subfamily, is also upregulated in the early stages of elicitor treatment [28,29]. Recently, a mechanism was elucidated in ATL31 and ATL6 ubiquitinate calcium-dependent protein kinase CPK28, fine-tuning the degradation of proteins and regulating the plant immune response [25]. In a previous report, it was shown that chitin elicitor rapidly and significantly increases the transcript levels of *ATL2* and *ATL6* genes, among other members of the ATL gene subfamily in *Arabidopsis* [21]. Chitin, found within the cell walls of fungi and the exoskeletons of insects and nematodes, functions as a stimulant for plant defense reactions [30]. Recent studies have shown that the cuticle mutant *eca2* (*expresión constitutiva de ATL2*) alters plant resistance to various pathogens, including *Botrytis cinerea*, *Pseudomonas syringae*, and *Spodoptera littoralis* insect herbivores [31]. However, the precise molecular mechanism and biological function of the ATL2 protein in response to fungal pathogen defense remain incompletely understood.

In this study, we aimed to elucidate the role of the ATL2 protein in the defense response against *A. brassicicola* fungal pathogen by manipulating its expression levels. Our findings indicate that ATL2, a RING-H2-type protein, exhibits typical E3 ubiquitin ligase activity, which is crucial for the defense response against *A. brassicicola* fungi. Further, using GFP tagging and cell fractionation analysis, we showed that ATL2 is localized to the plasma membrane. Moreover, we observed that exogenous chitin, a fungal cell wall component, significantly elevates the transcriptional levels and stability of the ATL2 protein, a process regulated by the ubiquitin/26S proteasome system.

## 2. Results

### 2.1. Arabidopsis ATL2 Functions as an E3 Ubiquitin Ligase That Is Localized to the Plasma Membrane

**Figure 1:**
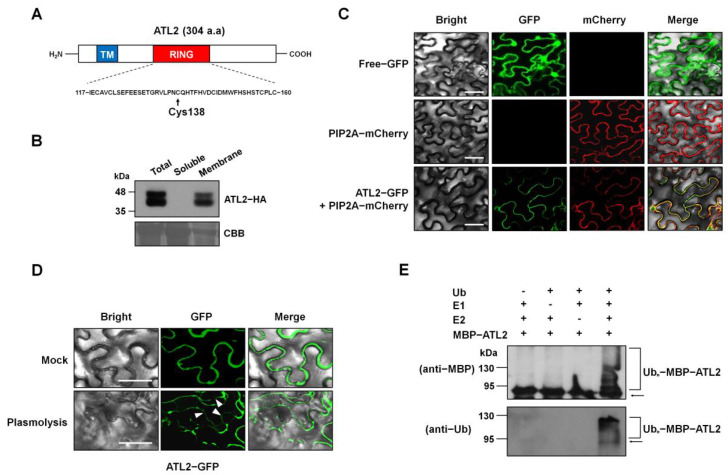
Illustration of the localization and activity of the ATL2 protein as an E3 ubiquitin ligase. (**A**) Schematic diagram of the ATL2 protein. The ATL2 protein contains a transmembrane domain (in blue, designated as TM) in the N-terminus and a RING-H2-type zinc finger motif (in red, designated as RING) in the middle region. The RING domain, consisting of 43 amino acids, contains an essential residue (Cys138) critical for E3 ubiquitin ligase activity, as indicated by the arrow. (**B**) Detection of ATL2 protein in the membrane fraction. The HA-tagged *ATL2*-overexpressing transgenic plant (*ATL2OX4-1*) was fractionated into soluble and membrane fractions from total extracts. The ATL2 protein was detected by using an anti-HA antibody (upper panel). Coomassie brilliant blue (CBB) was used as a loading control (lower panel). (**C**) Subcellular localization of ATL2-GFP via confocal laser scanning microscopy in *N. benthamiana*. The mCherry-fused AtPIP2A protein served as a plasma membrane marker. The “Merge” panel indicates the overlapped images of Bright, GFP (in green), and mCherry (in red) fluorescent signals. Scale bars = 50 µm. (**D**) Localization of ATL2-GFP fusion protein in *N. benthamiana* leaves before (upper panel) and after (lower panel) plasmolysis. The arrowheads in the lower panel indicate the location where the plasma membrane has separated from the cell wall. Scale bars = 50 µm. (**E**) E3 ubiquitin ligase activity of ATL2. The MBP-ATL2 fusion protein was assayed for E3 activity in the presence or absence of rabbit E1, human E2 (UbcH5b), and ubiquitin (Ub). The numbers on the left denote the molecular masses of marker proteins in kilodaltons (kDa). Samples were resolved by 7.5% (upper panel) and 12.5% (lower panel) SDS-PAGE. The anti-Ub antibody was used for detecting ubiquitinated proteins (lower panel), and the anti-MBP antibody was used for the detection of maltose fusion proteins (upper panel). Arrows indicate free MBP-ATL2 recombinant protein.

To gain a better understanding of the molecular function of the ATL2 protein, we utilized the Simple Modular Architecture Research Tool (SMART software version 9; http://smart.embl-heidelberg.de/ accessed on 1 January 2024) and DAS Transmembrane Prediction server (https://tmdas.bioinfo.se/ accessed on 1 January 2024) to predict potential functional domains. Based on these predictions, we identified a transmembrane domain and a RING-H2-type zinc finger motif in the N-terminus (30–57 a.a) and middle region (117–160 a.a) of the ATL2 protein (Figure 1A and Figure S1).

To investigate the subcellular localization of the ATL2 protein, we expressed the ATL2-HA protein in *Arabidopsis* whole plants and fractionated the cell extracts. Western blot analysis using an anti-HA monoclonal antibody revealed that the majority of the ATL2-HA protein was present in the membrane fraction, while it was undetectable in the soluble fraction (Figure 1B). To further confirm the membrane localization of the ATL2 protein, we fused a reporter gene encoding a green fluorescent protein (GFP) in frame with the *ATL2* coding region under the control of the *CaMV35S* promoter. We used AtPIP2A fused to mCherry as a plasma membrane marker [32] and observed the expression of the ATL2-GFP protein under a confocal microscope. Most of the ATL2-GFP signal co-localized with AtPIP2A-mCherry on the plasma membrane, while free GFP exhibited a diffused pattern in the cytoplasm of the *Nicotiana benthamiana* leaves (Figure 1C). In order to verify the localization of the ATL2 protein in the plasma membrane, we examined the localization of the ATL2-GFP protein during plasmolysis by treating the leaf tissue with 1 M mannitol for 20 min. After plasmolysis, we observed that GFP fluorescence was still visible in the membrane region, which had separated from the cell wall (Figure 1D).

The C_3_H_2_C_3_ (RING-H_2_)-type zinc finger motif of ATL2 is commonly associated with E3 ubiquitin ligase enzymes [15,33]. Previous research also demonstrated that the ATL2 protein interacts with components of the yeast ubiquitination machinery, providing further evidence that ATL2 may possess E3 ubiquitin ligase activity [20]. In light of these findings, we conducted an in vitro investigation of ATL2’s E3 ubiquitin ligase activity. We produced maltose-binding protein (MBP)-bound recombinant ATL2 (MBP-ATL2) using an *E. coli* expression system, and we purified it via affinity chromatography and tested its activity in the presence of ubiquitin, rabbit E1, and human E2 (ubcH5b). Our results demonstrate that MBP-ATL2 catalyzed the formation of poly-ubiquitin chains, indicating that ATL2 possesses E3 ubiquitin ligase activity (Figure 1E). Altogether, these findings suggest that the ATL2 protein functions as an E3 ubiquitin ligase integrated into the plasma membrane.

### 2.2. ATL2 Plays a Positive Role in the Defense Response against A. brassicicola Fungal Pathogen

Previous results from an ATH1 Affymetrix microarray analysis indicated a significant increase in the expression of various *A. thaliana ATL* genes, with a particular emphasis on *ATL2*, in response to chitin [34,35]. To validate this finding, we conducted quantitative RT-PCR analysis and histochemical *β*-glucuronidase (GUS) reporter experiments under both normal and chitin treatment conditions, covering various time intervals.

**Figure 2:**
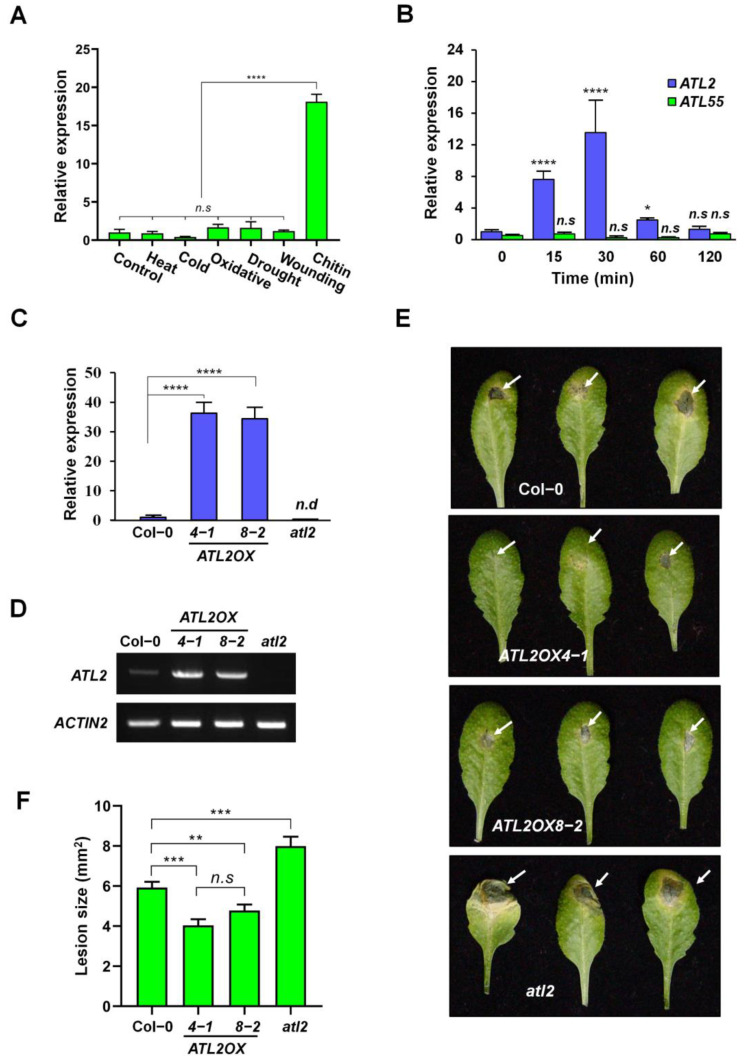
Disease resistance against *A. brassicicola* in *ATL2*-overexpressing plants (*ATL2OX4-1* and *8-2*) and the *atl2* mutant. (**A**) Real-time quantitative PCR (RT-qPCR) analysis of *ATL2* transcripts under various abiotic stresses and chitin treatments. Abiotic stresses and chitin treatments were applied to 10-day-old seedlings by incubating them in the following conditions—heat (37 °C), cold (4 °C), oxidative stress (100 µM MV; methyl viologen), drought (detached leaves were incubated on Whatman 3MM paper), wounding stress, or chitin (100 µg/mL)—under continuous light conditions for 1 h. (**B**) RT-qPCR analysis of *ATL2* transcripts after chitin treatments at various time points. Total RNA was extracted from 10-day-old plants and used for RT-qPCR analysis to investigate the transcript levels of *ATL2*. *ACTIN2* mRNA was used as a reference gene for data normalization. *ATL55* was included as a negative control. (**C**,**D**) Investigation of *ATL2* transcript levels in the *atl2* and *ATL2*-overexpressing plants with RT-qPCR (**C**) or RT-PCR (**D**) analysis. *ACTIN2* was used as a loading control for data normalization. Samples not detected are represented as “n.d”. (**E**) Photographs of leaves from Col-0, *ATL2OX4-1*, *ATL2OX8-2*, and *atl2* mutant post-infection with *A. brassicicola*. Leaves from the 6-week-old plant were detached and inoculated with 5 µL of *A. brassicicola* spores (approximately 5 × 10^5^ spores/mL). Images were captured 6 days post-inoculation, with white arrows indicating the regions that were inoculated. (**F**) The size of the lesion in *A. brassicicola* infected Col-0, *ATL2OX4-1, ATL2OX8-2*, and *atl2* plant leaves. The size of the lesions was quantified 6 days post-inoculation, and the data are presented as mean ± SD (*n* = 3). Significance levels are indicated as * *p* < 0.05, ** *p* < 0.005, *** *p* < 0.001, **** *p* < 0.0001. The *p*-value indicates the significance relative to the *A. brassicicola* treatment in wild-type plants, determined using GraphPad Prism 8 with two-way ANOVA followed by Dunnett’s multiple comparisons. These experiments were repeated three times as biological replicates, with similar results obtained in each repeat.

In non-treated plants, ATL2 exhibited ubiquitous expression in seedlings and various tissues, including stems, flowers, rosette leaves, cauline leaves, and roots. However, its expression was barely detectable (Figure S2). Notably, *ATL2* transcript levels were specifically induced by exogenous chitin treatment and remained largely unchanged under other stress conditions (Figure 2A). Further analysis revealed a rapid accumulation of *ATL2* transcripts at 15 min, peaking at 30 min with chitin treatment, whereas the expression of *ATL55* (a homolog of ATL2) showed no significant change during chitin treatment (Figure 2B and Figure S2C). These findings were substantiated by the observation of robust GUS activity driven by the *ATL2* promoter within 15 min of chitin treatment, compared to the control (Figure S2D). This strongly indicates that *ATL2* expression is promptly and specifically induced by chitin treatments.

Chitin is an elicitor of plant defense responses and is found in the cell walls of true fungi as well as the exoskeleton of insects and nematodes [30]. To investigate the role of the ATL2 protein in the fungal defense response, an ATL2 T-DNA insertion allele, *atl2* (*SALK_050772.54.50.x*), was obtained from the Arabidopsis Biological Resource Center (Figure S3A; upper panel). To confirm homozygous T-DNA insertion in the *ATL2* gene, the T-DNA junction region was amplified from genomic DNA via PCR using both a gene-specific forward primer and the T-DNA-specific reverse primer (Figure S3B). Additionally, *ATL2*-overexpressing plants were constructed under the control of the *CaMV35S* promoter (Figure S3A; lower panel). Two transgenic plants (*ATL2OX4-1* and *ATL2OX8-2*) were selected for further analysis as they showed significantly higher transcript levels with RT-PCR and RT-qPCR analysis compared to the wild-type plants (Figure 2C,D and Figure S3C,E). These analyses were also used to confirm that the *ATL2* transcript was not detectable in the *atl2* mutant, indicating that *atl2* is a knockout mutant (Figure 2C,D).

**Figure 3:**
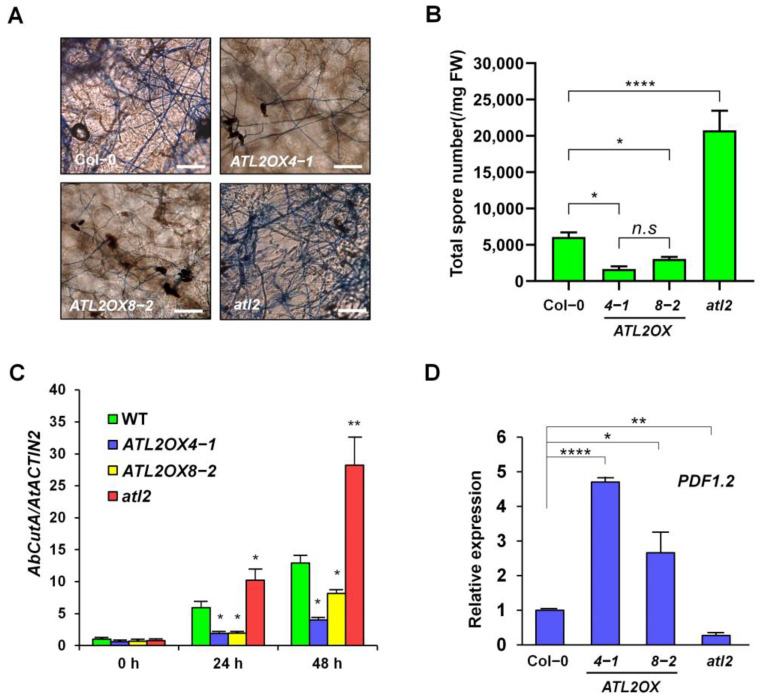
Investigation of *A. brassicicola* fungal pathogen defense. (**A**) Trypan blue staining showing fungal hyphae of *A. brassicicola* on *Arabidopsis* leaves; Col-0, *ATL2OX4-1*, *ATL2OX8-2*, and *atl2*. Scale bars = 50 µm. (**B**) Measurement of spore numbers in lesion. Total spores were collected from detached inoculated leaves and counted microscopically 6 days after inoculation. (**C**) The growth of the fungus in the inoculated plants was assessed by measuring the expression levels of *A. brassicicola CutinA* (*AbCutA*) relative to *A. thaliana ACTIN2*. Real-time quantitative PCR analysis was used to analyze *AbCutA* levels. Total DNA was extracted from *A. thaliana* Col-0, *ATL2OX4-1*, *ATL2OX8-2*, and *atl2* plants, which were inoculated with *A. brassicicola*, and the leaves were collected at 0, 24, and 48 h post-inoculation (hpi). These experiments were repeated three times (biological replicates) with similar results. (**D**) Analysis of *PDF1.2* expression levels in Col-0, *ATL2*-overexpressing plants, and *atl2* under normal growth conditions. Real-time quantitative PCR analysis was employed to monitor the expression of the defense-related gene *PDF1.2*. Total RNAs were extracted from 10-day-old plants, and the relative gene expression was calculated by comparing it with wild-type (Col-0). *ACTIN2* was used for data normalization in RT-qPCR analysis. The data are presented as mean ± SD (*n* = 3), and statistical significance is determined by * *p* < 0.05, ** *p* < 0.005, **** *p* < 0.0001. In panel B–D, the *p*-value indicated the significance relative to the wild-type plants and was determined and analyzed using GraphPad Prism 8 via two-way ANOVA followed by Dunnett’s multiple comparisons. These experiments were repeated three times (biological replicates) and showed similar results.

In order to investigate the role of ATL2 in the defense response against fungal pathogens, a spore suspension of *A. brassicicola* (approximately 5 × 10^5^ spores/mL) was dropped onto 6-week-old soil-grown *Arabidopsis* plants, including wild-type (WT) plants, two *ATL2*-overexpressing plants (*ATL2OX4-1* and *ATL2OX8-2*), and *atl2*, a knockout mutant of *ATL2*. The size of the fungal lesions was significantly increased in the *atl2* mutant and significantly reduced in the *ATL2*-overexpressing plants compared to WT plants (Figure 2E,F). To validate the findings, trypan blue staining was employed to examine the total spore count of *A. brassicicola* hyphae. Consistent with expectations, the hyphae of *A. brassicicola* exhibited intense staining in the *atl2* mutant, while dramatically reduced staining patterns were observed in the *ATL2*-overexpressing plants compared to the WT (Figure 3A,B). Additionally, the fungal growth was measured by quantifying the relative amounts of *A. brassicicola CutA* (*AbCutA*) DNA normalized to *A. thaliana ACTIN2* (*AtACTIN2*) DNA using qPCR amplification of DNA-inoculated leaves [36]. The fungal growth was considerably elevated in the *atl2* mutant but significantly reduced in the *ATL2*-overexpressing plants at 24 and 48 h post-inoculation by *A. brassicicola* (Figure 3C). To determine the involvement of *ATL2* expression in fungal pathogen defense, the transcription level of *PDF1.2* (*Plant defensin 1.2*; JA-responsive marker gene) was quantified with RT-qPCR analysis. The transcriptional expression of *PDF1.2* was significantly enhanced in the two *ATL2*-overexpressing plants (*ATL2OX4-1* and *ATL2OX8-2*) but was significantly reduced in the *atl2* mutant compared to that of the WT plants (Figure 3D). These results indicate that ATL2 is indispensable for fungal pathogen defense and its ectopic expression is adequate for promoting resistance to *A. brassicicola*.

### 2.3. The Defense against Fungal Pathogens Requires the E3 Ubiquitin Ligase Activity of ATL2

**Figure 4:**
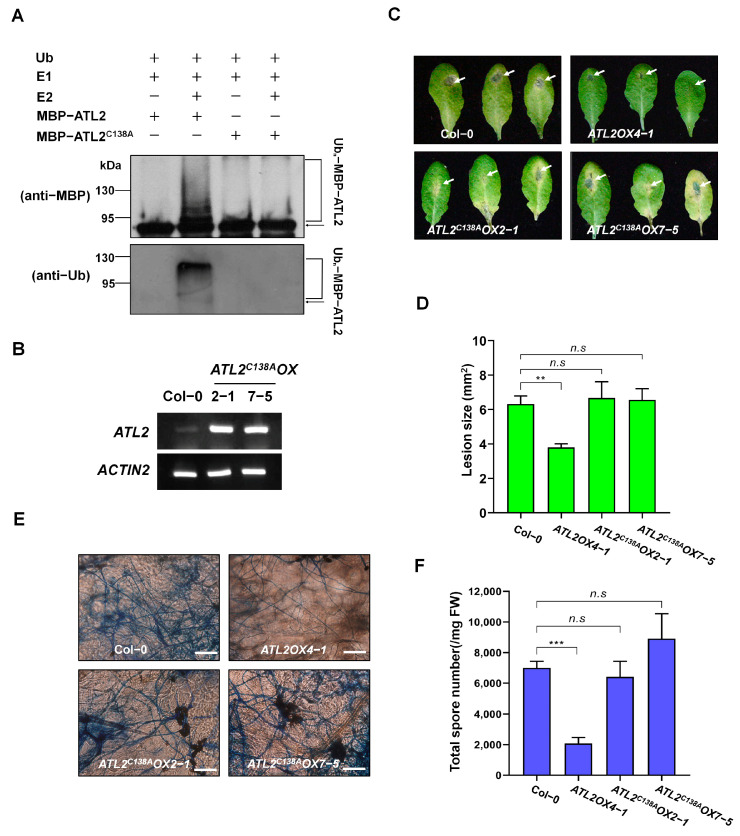
Requirement of ATL2 E3 ligase activity for disease response to *A. brassicicola*. (**A**) The activity of ATL2 E3 is dependent on the presence of the Cys138 residue in the RING domain. To analyze self-ubiquitination assays of MBP-ATL2 or MBP-ATL2^C138A^ proteins in the presence of rabbit E1, human E2, and Ub, Western blot analyses were conducted using anti-MBP and anti-Ub antibodies. Arrows denote the non-ubiquitinated forms of the proteins. (**B**) RT-PCR analysis of *ATL2* transcripts of Col-0, *ATL2^C138A^OX2-1-*, and *ATL2^C138A^OX7-5*-overexpressing plants. *ACTIN2* was used as a loading control. (**C**) The presence of the C138A mutation in the RING domain of ATL2 is necessary for a disease response to *A. brassicicola*. Photographs of the leaves of Col-0, *ATL2OX4-1-*, *ATL2^C138A^OX2-1-*, and *ATL2^C138A^OX7-5*-overexpressing plants post-infection with *A. brassicicola* are shown. Detached leaves from 6-week-old plants were inoculated with a 5 µL droplet of *A. brassicicola* spores (approximately 5 × 10^5^ spores/mL), and photographs were taken 6 days after inoculation. The white arrows indicate inoculated regions. (**D**) Size of lesions in *A. brassicicola* infected Col-0, *ATL2OX4-1-*, *ATL2^C138A^OX2-1-*, and *ATL2^C138A^OX7-5*-overexpressing plant leaves. Lesion size was measured at 6 days after inoculation. (**E**) Trypan blue staining showing fungal hyphae of *A. brassicicola* on *Arabidopsis* leaves; Col-0, *ATL2OX4-1*, *ATL2^C138A^OX2-1*, and *ATL2^C138A^OX7-5*. Scale bars = 50 µm. (**F**) Measurement of spore numbers in lesions. The spore numbers in lesions were measured by collecting total spores from detached inoculated leaves and counting them microscopically at 6 days after inoculation. The data are shown as mean ± SD (*n* = 3), with ** *p* < 0.005, and *** *p* < 0.001 indicating the significance level. In panels D and F, the *p*-value indicates the significance relative to the *A. brassicicola* treatment at wild-type (Col-0) plants and was determined and analyzed using GraphPad Prism 8 via two-way ANOVA followed by Dunnett’s multiple comparison. n.s represents not significant. These experiments were repeated three times (biological replicates) with similar results.

Previous research showed that the third cysteine in the RING motif of ATL proteins plays a crucial role in their E3 ligase activity [29,37]. Based on this knowledge, Cys138 in ATL2 is predicted to be important for E3 ubiquitin ligase activity (Figure 1A). A mutated construct called ATL2^C138A^ was created to confirm this by substituting the Cys138 with Ala using site-directed mutagenesis. This construct was then purified via affinity chromatography and subjected to an in vitro ubiquitination assay to test its E3 ligase activity. Results showed that the presence of MBP-ATL2^C138A^ completely abolished E3 ubiquitin ligase activity, whereas the wild-type ATL2 demonstrated strong activity (Figure 4A). This suggests that Cys138 is indeed essential for E3 ubiquitin ligase activity in ATL2. To determine the importance of ATL2’s ubiquitin ligase activity in the defense response against fungal pathogens, mutated *ATL2*-overexpressing plants were created using site-directed mutagenesis. These plants carried a Cys138Ala mutation in the RING zinc finger motif and were under the control of the *CaMV35S* promoter.

Two transgenic plants, *ATL2^C138A^OX2-1* and *ATL2^C138A^OX7-5*, were selected for further analysis among seven independent transgenic plants obtained (Figure S3D,E) due to their significantly higher transcript levels as shown via RT-qPCR analysis (Figure 4B and Figure S3D,E). To test their resistance to the *A. brassicicola* fungal pathogen, fungal spore suspensions (approximately 5 × 10^5^ spores/mL) were dropped onto six-week-old soil-grown wild-type (Col-0), *ATL2OX4-1*, *ATL2^C138A^OX2-1*, and *ATL2^C138A^OX7-5* transgenic plants. *ATL2OX4-1* plants showed increased resistance to *A. brassicicola* compared to WT plants. However, *ATL2^C138A^OX2-1* and *ATL2^C138A^OX7-5* transgenic plants exhibited similar resistance to the *A. brassicicola* fungal pathogen as WT plants (Figure 4C). Additionally, *ATL2OX4-1* showed a reduction in both the number of spores and lesion size, while *ATL2^C138A^OX2-1* and *ATL2^C138A^OX7-5* showed similar lesion size and total spore numbers to WT plants after treatment with *A. brassicicola* (Figure 4D,F). Moreover, staining patterns revealed that the hyphae of *A. brassicicola* were significantly reduced in *ATL2OX4-1* but not in *ATL2^C138A^OX2-1* and *ATL2^C138A^OX7-5* compared to WT plants (Figure 4E). These findings strongly suggest that E3 ubiquitin ligase activity of ATL2 is required for the defense response against the *A. brassicicola* fungal pathogen.

### 2.4. The Stability of ATL2 Protein Is Regulated by Treatments with 26S Proteasome Inhibitor and Chitin

**Figure 5:**
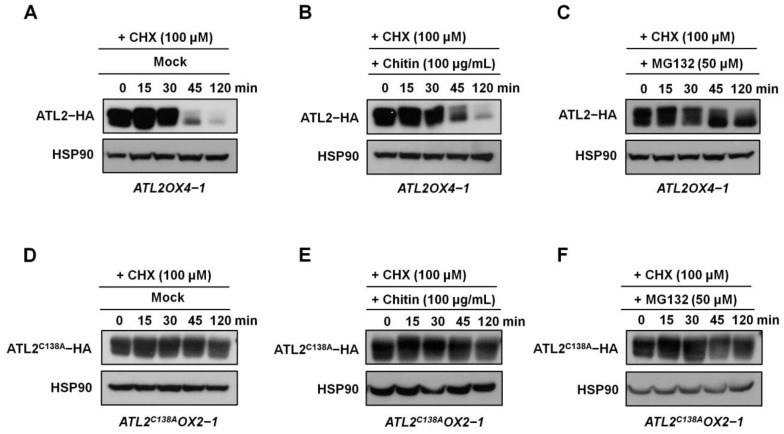
Protein stability of ATL2. Total proteins were extracted from 10-day-old *ATL2OX4-1-* (**A**–**C**) and *ATL2^C138A^OX2-1*-overexpressing plants (**D**–**F**), which were then treated with cycloheximide (100 µM) with or without chitin (100 µg/mL), or with or without the 26S proteasome inhibitor (MG132) for the indicated time points. Western blot analyses were performed using an anti-HA antibody. HSP90 was used as a loading control. The above experiments were repeated twice (biological replicates) and yielded similar results.

**Figure 6:**
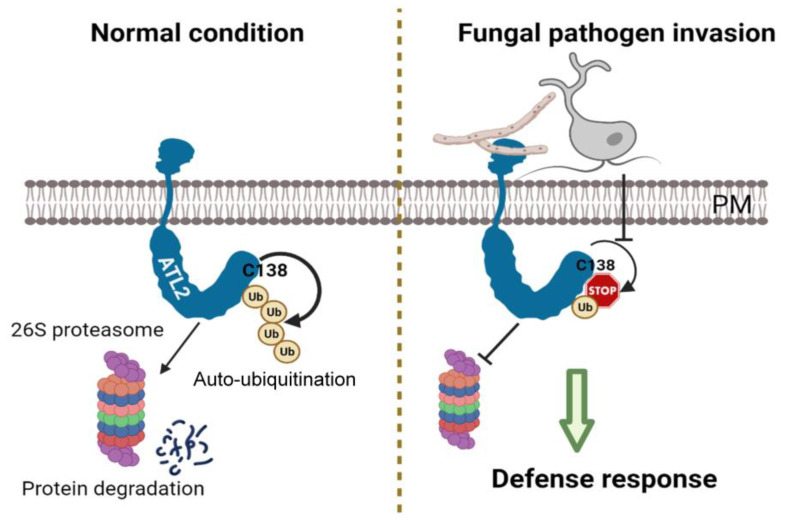
Hypothetical model of ATL2 in fungal pathogen defense response. Under normal growth conditions, the plasma-membrane-localized ATL2 E3 ligase is self-regulated through auto-ubiquitination in the 26S proteasome mechanism. However, *ATL2* transcripts show a significant increase upon fungal pathogen invasion and stabilize the ATL2 protein. Consequently, ATL2 plays a positive role in necrotrophic fungal pathogen defense responses. This figure was created using BioRender (https://biorender.com/).

To investigate the effect of chitin treatment on the stability of ATL2 protein and whether it undergoes degradation by the 26S proteasome, we used HA-tagged *ATL2OX4-1* and *ATL2^C138A^OX2-1* transgenic plants, controlled by the constitutive *CaMV35S* promoter. Ten-day-old seedlings were treated with cycloheximide (CHX) to inhibit de novo protein synthesis, and the stability of the ATL2 protein was analyzed using Western blotting with an anti-HA antibody, with or without the 26S proteasome inhibitor (MG132, Sigma-Aldrich, Saint Louis, MO, USA) or chitin treatments. The results showed that under CHX treatment conditions, the ATL2-HA protein degraded rapidly (Figure 5A). Interestingly, the degradation of the ATL2-HA protein was significantly delayed by chitin and MG132 treatments (Figure 5B,C), indicating that the ubiquitin/26S proteasome pathway mediates the degradation of the ATL2 protein and that chitin significantly delays its degradation. Furthermore, we examined the role of E3 ligase activity in regulating the stability of the ATL2 protein by analyzing the stability of mutated ATL2-HA (ATL2^C138A^-HA) protein in *ATL2^C138A^OX2-1* transgenic plants (Figure S4A). Transcript levels of *ATL2-HA* were similar to *ATL2^C138A^-HA* under normal growth conditions (Figure S4B), but the quantity of mutated ATL2-HA protein was significantly increased in *ATL2^C138A^-HA* transgenic plants (Figure S4A). Interestingly, the degradation of the ATL2^C138A^-HA protein was significantly delayed via CHX treatment, as well as chitin and MG132 treatments (Figure 5D–F), suggesting that the stability of the ATL2 protein is regulated by self-ubiquitination and is highly dependent on the integrity of the E3 ubiquitin ligase activity of the 138 cysteine residue in the RING motif. We also investigated whether the C138A mutation affects the localization of the protein to the plasma membrane by fusing a reporter gene encoding the green fluorescent protein (GFP) in frame with the ATL2^C138A^ coding region under the control of the *CaMV35S* promoter. The results showed that both ATL2-GFP and ATL2^C138A^-GFP were able to localize to the plasma membrane (Figure S4C). These results demonstrate that chitin treatment delays the degradation of the ATL2 protein through the ubiquitin/26S proteasome pathway and that the E3 ubiquitin ligase activity of the 138 cysteine residue in the RING motif plays a critical role in regulating its stability. In summary, our findings suggest that ATL2 positively regulates defense against fungal pathogens, with its E3 ubiquitin activity being essential for this response, highlighting the crucial role of self-ubiquitination of ATL2 in regulating plant immunity (Figure 6).

## 3. Discussion

### 3.1. The ATL2 Induced with Chitin Is Essential for the Early Response to Fungal Pathogen Defense

The ubiquitin/26S proteasome system, along with its associated E3 ubiquitin ligases, is crucial in disease resistance mechanisms [38,39,40]. The ATL family of E3 ligases is particularly involved in the response to pathogen defense. In *Arabidopsis*, the expression of *ATL6* is rapidly and significantly increased upon flg22 treatment [41]. Similarly, tomato *LeATL6* is expressed in cell wall protein fractions and is influenced by jasmonic acid signaling cascades. ATL6 and ATL31 share structural similarities, and their transcript levels are induced via PAMP treatment, resulting in enhanced resistance to the *Pst*DC3000 bacterial pathogen [27,41]. Another PAMP-responsive gene, *ATL9*, also possesses E3 ubiquitin ligase activity and shares structural similarities with ATL6. In *Arabidopsis*, *atl9* T-DNA insertional mutants exhibit a reduced expression of *PDF1.2*, *PCC1*, and *FBS1*, and they exhibit an increased susceptibility to *Golovinomyces cichoracearum* biotrophic powdery mildew, while the ectopic expression of *ATL9* transgenic plants displayed increased resistance to the same pathogen [42]. In rice, *EL5*, a structural homolog of the ATL family, is rapidly induced upon treatment with oligosaccharides, eliciting a defense response in suspension-cultured rice cells within 30 min [29].

In previous studies, *Arabidopsis* ATL2 was identified as a chitin-induced gene via microarray analysis and was suggested to be involved in the response to fungal pathogen defense, supported by the analysis of *eca* mutants displaying ectopic expression of *ATL2*. Recently, the Catherine group reported that the *eca2* mutant (*expresión constitutiva de ATL2*) displayed enhanced resistance to *B. cinerea*, *P. syringae*, and the generalist herbivorous insect *S. littoralis*. However, while the disease resistance of *eca2* was significantly increased, the disease resistance of other *eca* mutants (e.g., *eca1*, *eca3*, *eca4*) was not significantly different from that of the wild-type plants despite the increase in *ATL2* gene expression, leading to controversy about the role of ATL2 in disease resistance. In our study, we confirmed that the transcript level of *ATL2* increased rapidly within 15 min of chitin fungal elicitor treatment and reached its maximum at 30 min after treatment (Figure 2B and Figure S2C). Additionally, the stability of ATL2 protein greatly increased with chitin treatment (Figure 5). *ATL2*-overexpressing plants displayed greatly enhanced resistance to the *A. brassicicola* fungal pathogen (Figure 2). These results indicate that ATL2 plays an indispensable role in response to necrotrophic pathogens by rapidly and highly elevating *ATL2* transcript levels and its protein stability. Therefore, we have experimentally confirmed that ATL2 has an important function in fungal resistance.

### 3.2. ATL2 Is a Typical RING H2-Type Ubiquitin Ligase and Plays an Essential Role in Plant Defense Responses

The *Arabidopsis* genome encodes 469 members that harbor one or more conserved RING domains, which are classified as E3 ubiquitin ligases due to their consensus amino acid sequence containing Cys and His residues that coordinate with zinc ions for activity [15]. The ATL subfamily is part of the RING-type E3 ubiquitin ligase family. It consists of 91 members with a putative transmembrane domain located at the N-terminus and the RING-H2-type zinc finger motif in the middle or C-terminus [43]. ATL2 contains a highly conserved RING-H2-type zinc finger motif (Cys-X_2_-Cys-X_15_-Cys-X_1_-His-X_2_-His-X_2_-Cys-X_10_-Cys-X_2_-Cys; C_3_H_2_C_3_) in the middle region. Additionally, the Guzman group reported that ATL2 protein interacts with components of yeast ubiquitination machinery and is predicted to be an E3 ubiquitin ligase [20]. Our study demonstrates that ATL2 possesses E3 ubiquitin ligase activity in vitro, and that a mutation in the third conserved Cys138 residue in the RING domain of ATL2 abolishes this activity (Figure 4A). ATL2 is a typical RING-H2-type zinc finger protein that functions as an E3 ubiquitin ligase in planta via the ubiquitin/26S proteasome pathway. In an earlier study, it was observed that the overexpression of pepper CaRING1, an E3 ubiquitin ligase, resulted in a cell death phenotype [44]. However, overexpression of an inactive CaRING1 with a single mutation in the RING motif led to a decrease in cell death and early defense responses [44]. This suggests that the E3 ligase activity of CaRING1 is essential for cell death and the SA-dependent disease response in *Capsicum annuum* [44]. To investigate whether the E3 ubiquitin ligase activity of ATL2 is necessary for defense against fungal pathogens, we utilized the plants overexpressing an inactive E3 ubiquitin ligase form of ATL2 (ATL2^C138A^). Our results demonstrated that plants overexpressing the active ATL2 were highly resistant to a necrotrophic pathogen, whereas plants overexpressing the inactive ATL2^C138A^ showed a defense response similar to that of wild-type plants (Figure 4). This finding supports the idea that the ubiquitin ligase activity of ATL2 is required for proper regulation of the defense response against fungal pathogens in *Arabidopsis*.

### 3.3. ATL2 Protein Stability Is Regulated by Self-Ubiquitination

Ubiquitination is a process involved in targeting proteins for degradation through the 26S proteasome pathway. RING-type ubiquitin ligases, like ATL55/RING1, have a notable feature of self-ubiquitination in vitro and in vivo [45,46]. ATL55/RING1 is essential for the regulation of programmed cell death in plants and regulates its stability by self-ubiquitination [47]. Similarly, to determine if ATL2 is regulated by self-ubiquitination and degraded by the 26S proteasome system, we treated *ATL2*-overexpressing plants with the MG132 proteasome inhibitor and chitin. The results indicated that the stability of the ATL2 protein is significantly increased by these treatments, which is dependent on the E3 ubiquitin ligase activity of ATL2. This suggests that the fungal pathogen stabilizes the ATL2 protein, and the degradation of ATL2 by the ubiquitin/26S proteasome system is gradually delayed during a defense response (Figure 5). Our study also found that the ATL2 protein is localized to the plasma membrane and has typical RING-H2-type E3 ubiquitin ligase activity (Figure 1). The transcript level and stability of the ATL2 protein are greatly increased under exogenous chitin treatments (Figure 2 and Figure 5). These findings indicate that ATL2 positively regulates fungal pathogen defense and that its E3 ubiquitin activity is essential for fungal pathogen defense responses. Therefore, our results suggest that the self-ubiquitination of ATL2 plays an important role in the regulation of plant immunity against fungal pathogens (Figure 6). Recently, ATL6 and ATL31 were demonstrated to interact with the CPK28 substrate, regulating this protein by inducing the ubiquitination of CPK28, which is related to the defense mechanism against bacterial pathogens. Our mission going forward is to identify the substrate(s) of ATL2 and to fully understand which proteins are regulated and ubiquitinated by ATL2 E3 ubiquitin ligase for the precise mechanism used in response to fungal pathogen defense.

## 4. Materials and Methods

### 4.1. Plant Materials and Growth Conditions

*A. thaliana* ecotype Columbia (Col-0), two *ATL2*-overexpressing plants (*ATL2OX4-1*, *ATL2OX8-2*), two *ATL2^C138A^*-overexpressing plants (*ATL2^C138A^OX2-1* and *ATL2^C138A^OX7-5*), and a T-DNA inserted mutant, SALK_050772.54.50.x (*atl2*), were used in this study. The seeds were surface-sterilized using a vapor-phase sterilization method [48,49]. Approximately 10 µL volumes of seeds were transferred to 1.5 mL micro-centrifuge tubes. These tubes were placed into a desiccator jar containing a beaker with 100 mL of bleach and 3 mL of concentrated HCl; then, they were sterilized using chlorine fumes for 4 h. The sterilized seeds were then sown onto agar plates containing one-half-strength Murashige and Skoog (MS) medium, 2% (*w*/*v*) sucrose, and 0.25% (*w*/*v*) phytagel, pH 5.7. The plates were kept in the dark at 4 °C for 3 days for stratification before being transferred to a growth chamber for germination under long-day conditions (16 h of light/8 h of darkness cycle) at 21 °C with a light intensity of approximately 120 µE m^−2^ s^−1^. For seed harvest, the plants were grown under the same conditions. However, plants were also grown under short-day conditions (8 h of light/16 h of darkness cycle) for fungal pathogen infection with *A. brassicicola*.

### 4.2. RT-PCR and RT-qPCR Analyses under Various Abiotic Stresses and Chitin Treatments

Ten-day-old seedlings were placed under abiotic stresses and chitin treatments under continuous light conditions as follows: incubation at 37 °C (for heat) and/or at 4 °C (for cold), addition of 100 µM MV (methyl viologen; for oxidative stress), dehydration on Whatman 3MM paper (for drought), exposure to wounding stress, and treatment with 100 µg/mL of chitin (Sigma-Aldrich, Saint Louis, MO, USA) [50]. Following specified treatment durations, the plants were promptly frozen in liquid nitrogen. For reverse transcription, Superscript III (Invitrogen, Waltham, MA, USA) was utilized on two microgram aliquots of total RNA extracted from wild-type or mutant seedlings. The resulting cDNA served as the template for subsequent PCR reactions, with *ACTIN2* employed as a quantifying control. Real-time quantitative RT-PCR (RT-qPCR) analysis was carried out using the CFX96 Touch™ Real-Time PCR Detection System (Bio-Rad, Hercules, CA, USA), employing the QuantiMix SYBR Kit (PKTechnology, Wichita, Kansas) and primers ranging from 100 to 300 bp designed to eliminate genomic DNA contamination. The *ACTIN2* gene functioned as an internal control for normalizing gene expression across different samples. The primer sequences can be found in Table S1.

### 4.3. Histochemical Analysis of β-Glucuronidase (GUS) Activity

The histochemical staining protocol for evaluating GUS activity in transgenic plants was performed as outlined by Jefferson et al. in 1987 [51,52]. Tissues were immersed in a staining solution comprising 50 mM sodium phosphate (pH 7.0), 10 mM EDTA, 1 mM 5-bromo-4-chloro-3-indoyl glucuronide, 0.2 mM potassium ferricyanide, and 0.2 mM potassium ferrocyanide, and were then incubated at 37 °C for 2 h. Subsequent to the staining process, the tissues underwent a series of ethanol washes (10%, 30%, 50%, 70%, and 100%) to eliminate chlorophyll and were prepared for observation and photography.

### 4.4. Fungal Culture and Disease Assay

The *A. brassicicola* strain *MUCL20297* was cultured on potato dextrose agar for a period of 10 days at 25 °C. The resultant spores were then collected and suspended in a solution containing 0.025% (*v*/*v*) Tween-20. For the inoculation of 6-week-old plants, a 5 µL droplet of the spore suspension, containing approximately 5 × 10^5^ spores/mL, was applied to each leaf. Subsequently, the inoculated leaves were covered with a transparent shield to maintain high humidity. After a 6-day incubation period, the diameter of each lesion was measured. To determine spore count, 10 leaves were gathered, and a spore-containing suspension was prepared by vigorously shaking them in 5 mL of 0.025% Tween-20 in a Falcon tube. After removing the leaves, the spores were isolated by centrifuging the suspension at 5000× *g* for 10 min. The spores were then resuspended in 200 µL of 0.025% Tween-20 solution, serially diluted, and counted using a light microscope. For the visualization of fungal hyphae, infected leaves collected 6 days post-inoculation were stained according to a previously established protocol [35]. The detached leaves were stained with a 250 µg/mL trypan blue solution, prepared by mixing lactic acid, distilled water, and glycerol at a 1:1:1 ratio. After staining for 15 min, the samples were mounted in 60% (*v*/*v*) glycerol and observed using a light microscope.

### 4.5. Expression and Purification of the Recombinant Proteins

The process of expressing and purifying the recombinant proteins involved using *E. coli* strain *BL21 (DE3) pLyS* per the manufacturer’s instructions from NEB (NEB, Hitchin, UK) [53]. The *pIH1119* vector was utilized to generate the MBP-ATL2 and MBP-ATL2^C138A^ recombinant proteins. In a 50 mL volume of LB media, the MBP-ATL2 and MBP-ATL2^C138A^ recombinant proteins were expressed by growing transformed cells of *E. coli* strain *BL21* (*DE3*) *pLyS* at 37 °C until reaching an OD_600_ of 0.4 to 0.6. To induce protein expression, 1.0 mM IPTG was added to the LB media and incubated for 4 h at 30 °C. Afterward, the cells were collected via centrifugation (6000× *g* for 15 min) and sonicated in 2 mL of lysis buffer containing 137 mM NaCl, 2.7 mM KCl, 10 mM Na_2_HPO_4_, 2 mM KH_2_PO_4_, pH 7.4, 1 mM PMSF, 1 mM DTT, 1% (*v*/*v*) Triton X-100 (Sigma-Aldrich, St. Louis, MO, USA). The lysates were then centrifuged at 12,000× *g* for 15 min, and the supernatants were applied to an amylase resin affinity column (NEB, Ipswich, MA, USA). Finally, the MBP fusion recombinant proteins were eluted using an elution buffer with 10 mM maltose.

### 4.6. In Vitro Ubiquitination Assay

The in vitro ubiquitination assays were conducted following the procedure outlined by Zhang et al. in 2005 [54]. Purified MBP-ATL2 and MBP-ATL2^C138A^ recombinant proteins (200 ng each) were mixed with 0.1 µg rabbit E1, 0.2 µg E2 UbcH5b, and 10 µg ubiquitin in a reaction buffer (50 mM Tris-HCl, pH 7.4, 5 mM MgCl_2_, 2 mM ATP, 2 mM DTT) and incubated for 2 h. The reaction was terminated by adding 2 × SDS-PAGE sample buffer and heating at 100 °C for 5 min. Fifteen microliters of the reaction mixture were separated via electrophoresis on 7.5% and 12.5% SDS-PAGE gels, and the ubiquitinated proteins were detected with Western blotting using anti-MBP and anti-Ub antibodies.

### 4.7. Cell Fractionation Assay

Cell fractionation analysis was conducted on entire seedlings of HA-tagged *ATL2-OX4-1* transgenic plants. The tissue was ground in liquid nitrogen and suspended in an extraction buffer (50 mM Tris-MES, pH 8.0, 0.5 M sucrose, 1 mM MgCl_2_, 10 mM EDTA, 10 mM EGTA, 10 mM ascorbic acid, 5 mM DTT, protease inhibitor cocktail; Roche, Basel, Switzerland) on ice, using a previously outlined method [55]. The resulting total protein extracts were centrifuged at 10,000× *g* for 10 min, and the supernatant was recovered and subjected to centrifugation at 125,000× *g* for 1 h to separate the membrane fraction from insoluble materials. The membrane fraction was reconstituted in a detergent-free buffer (5 mM potassium phosphate (pH 7.8), 2 mM DTT, with protease inhibitor cocktail; Roche). Portions of each sample were combined with a SDS-PAGE loading buffer for protein gel analysis. SDS gel electrophoresis was used to separate total proteins, and Western blots were probed with anti-HA monoclonal antibodies (Roche, Basel, Switzerland). Coomassie brilliant blue R250 (CBB) was applied to stain the gels for use as a loading control.

### 4.8. Subcellular Localization and Plasmolysis

The N-terminal end of GFP in the *pMDC83* vector was subcloned with the PCR DNA fragment obtained by amplifying the coding region of *ATL2*. For the transient expression of GFP-fused ATL2, the subcellular localization construct was introduced into *Agrobacterium tumefaciens* strain *GV3101* along with the plasma membrane marker AtPIP2A [32,56]. Three- to four-week-old *N. benthamiana* leaves were infiltrated, and images were recorded after 2 days. Plasmolyzed leaf samples were prepared by immersing them in 1.0 M mannitol for 20 min, and the leaves were examined under an Olympus FV1000 confocal laser scanning microscope (Olympus Corporation, Tokyo, Japan). The GFP signal was excited with a 488 nm wavelength and the emission spectra was recorded between 510 and 540 nm, whereas the mCherry signal was excited with a 585 nm wavelength, and emission spectra were collected between 605 nm and 650 nm using an Argon ion laser system. Fluorescence analysis of the images was performed using Olympus FluoView software (version 01.07.01.00).

### 4.9. MG132, Cycloheximide, and Chitin Treatments

*Arabidopsis* seedlings expressing the HA-tagged ATL2 or ATL2^C138A^ protein under the regulation of the *CaMV35S* promoter were germinated and cultivated on selective media for 10 days (with a light/dark cycle of 16:8 h). Following this, the seedlings were transferred to half-strength MS liquid medium containing MG132 (Sigma-Aldrich, Saint Louis, MO, USA) and/or cycloheximide (Sigma-Aldrich, Saint Louis, MO, USA), and/or chitin (Sigma-Aldrich, Saint Louis, MO, USA) at different concentrations. The treated seedling samples were collected at predetermined times for subsequent protein expression analysis using immunoblotting. In immunoblotting, the seedlings underwent extraction in a denaturing buffer—50 mM Tris/HCl pH 6.8, 4% (*w*/*v*) SDS, 2% (*v*/*v*) 2-mercaptoethanol, 10% (*v*/*v*) glycerol, and 0.001% (*w*/*v*) bromophenol blue—at a ratio of 20 volumes (*w*/*v*). Following a 3 min incubation at 100 °C, the extracts were filtered via centrifugation at 8000× *g* for 3 min. The total proteins were then separated via SDS gel electrophoresis, and Western blots were subjected to probing with the anti-HA monoclonal antibody (Roche, Basel, Switzerland). Coomassie brilliant blue R250 was utilized to stain the gels, and HSP90 (90 kDa) served as the designated loading control.

## Data Availability

The authors declare that all other data supporting the findings of this study are available within the manuscript and its supplementary files or are available from the corresponding author upon request.

## Supplementary Materials

The following supporting information can be downloaded at: https://www.mdpi.com/article/10.3390/ijms25042388/s1.
