# Correction: Kim et al. The Auto-Regulation of ATL2 E3 Ubiquitin Ligase Plays an Important Role in the Immune Response against *Alternaria brassicicola* in *Arabidopsis thaliana*. *Int. J. Mol. Sci.* 2024, *25*, 2388

**DOI:** 10.3390/ijms251810095

**Published:** 2024-09-20

**Authors:** Daewon Kim, Su Jeong Jeon, Jeum Kyu Hong, Min Gab Kim, Sang Hee Kim, Ulhas S. Kadam, Woe-Yeon Kim, Woo Sik Chung, Gary Stacey, Jong Chan Hong

**Affiliations:** 1 Division of Life Science and Division of Applied Life Science (BK21 Four), Plant Molecular Biology and Biotechnology Research Center (PMBBRC), Gyeongsang National University, 501 Jinju-daero, Jinju 52828, Republic of Korea; kimdaew@missouri.edu (D.K.); sujeongjeon83@gmail.com (S.J.J.); sangheekim@gnu.ac.kr (S.H.K.); ukadam@gnu.ac.kr (U.S.K.);; 2 Division of Plant Science & Technology, C.S. Bond Life Sciences Center, University of Missouri, Columbia, MO 65211, USA; staceyg@missouri.edu; 3 Laboratory of Horticultural Crop Protection, Division of Horticultural Science, Gyeongsang National University, 33 Dongjin-ro, Jinju 52725, Republic of Korea; jkhong@gnu.ac.kr; 4 Agri-Food Bio Convergence Institute, Gyeongsang National University, 33 Dongjin-ro, Jinju 52725, Republic of Korea; 5 College of Pharmacy and Research Institute of Pharmaceutical Science, Gyeongsang National University, 501 Jinju-daero, Jinju 52828, Republic of Korea; mgk1284@gnu.ac.kr; 6 Division of Applied Life Science (BK21 Four), Plant Biological Rhythm Research Center (PBRRC), Plant Molecular Biology and Biotechnology Research Center (PMBBRC), Gyeongsang National University, 501 Jinju-daero, Jinju 52828, Republic of Korea

In the original publication [1], there was a mistake in Figure 4 as published. The Col-0 image in Figure 4C was mistakenly duplicated as the *ATL2^C138A^OX7-5* image. The corrected Figure 4 appears below. The authors state that the scientific conclusions are unaffected. This correction was approved by the Academic Editor. The original publication has also been updated.

## Figures and Tables

**Figure 4:**
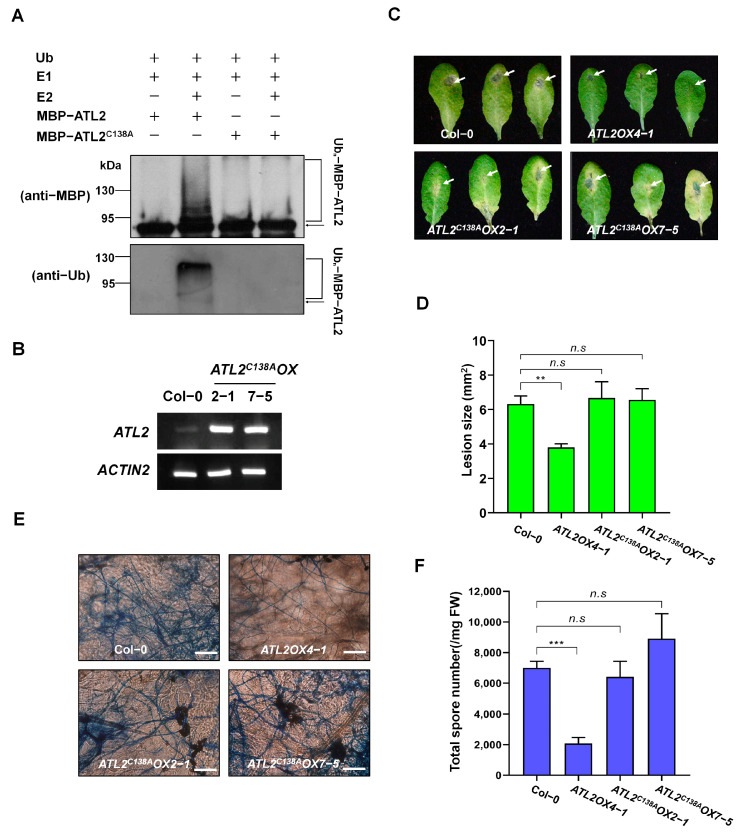
Requirement of ATL2 E3 ligase activity for disease response to *A. brassicicola*. (**A**) The activity of ATL2 E3 is dependent on the presence of the Cys138 residue in the RING domain. To analyze self-ubiquitination assays of MBP-ATL2 or MBP-ATL2^C138A^ proteins in the presence of rabbit E1, human E2, and Ub, Western blot analyses were conducted using anti-MBP and anti-Ub antibodies. Arrows denote the non-ubiquitinated forms of the proteins. (**B**) RT-PCR analysis of *ATL2* transcripts of Col-0, *ATL2^C138A^OX2-1-*, and *ATL2^C138A^OX7-5*-overexpressing plants. *ACTIN2* was used as a loading control. (**C**) The presence of the C138A mutation in the RING domain of ATL2 is necessary for a disease response to *A. brassicicola*. Photographs of the leaves of Col-0, *ATL2OX4-1-*, *ATL2^C138A^OX2-1-*, and *ATL2^C138A^OX7-5*-overexpressing plants post-infection with *A. brassicicola* are shown. Detached leaves from 6-week-old plants were inoculated with a 5 µL droplet of *A. brassicicola* spores (approximately 5 × 10^5^ spores/mL), and photographs were taken 6 days after inoculation. The white arrows indicate inoculated regions. (**D**) Size of lesions in *A. brassicicola* infected Col-0, *ATL2OX4-1-*, *ATL2^C138A^OX2-1-*, and *ATL2^C138A^OX7-5*-overexpressing plant leaves. Lesion size was measured at 6 days after inoculation. (**E**) Trypan blue staining showing fungal hyphae of *A. brassicicola* on *Arabidopsis* leaves; Col-0, *ATL2OX4-1*, *ATL2^C138A^OX2-1*, and *ATL2^C138A^OX7-5*. Scale bars = 50 µm. (**F**) Measurement of spore numbers in lesions. The spore numbers in lesions were measured by collecting total spores from detached inoculated leaves and counting them microscopically at 6 days after inoculation. The data are shown as mean ± SD (*n* = 3), with ** *p* < 0.005, and *** *p* < 0.001 indicating the significance level. In panels D and F, the *p*-value indicates the significance relative to the *A. brassicicola* treatment at wild-type (Col-0) plants and was determined and analyzed using GraphPad Prism 8 via two-way ANOVA followed by Dunnett’s multiple comparison. n.s represents not significant. These experiments were repeated three times (biological replicates) with similar results.
