# Supplementary material for: The Auto-Regulation of ATL2 E3 Ubiquitin Ligase Plays an Important Role in the Immune Response against Alternaria brassicicola in Arabidopsis thaliana
Source: Int J Mol Sci. 2024 Feb 17;25(4):2388. doi: 10.3390/ijms25042388 (PMC10889567; doi:10.3390/ijms25042388)
Supplement: Supplementary file 1 [file ijms-25-02388-s001.zip › ijms-2863681-supplementary.pdf]

SUPPORTING INFORMATION

A

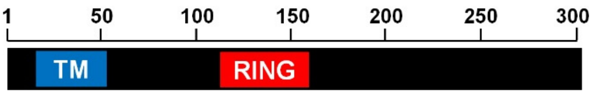

B

```
  1 MNSNDQDPIP FRPEDNNFSG SKTYAMSGKI MLSAIVILFF VVILMVFLHL
 51 YARWYLLRAR RRHLRRRSRN RRATMVFFTA DPSTAATSVV ASRGLDPNVI
101 KSLPVFTFSD ETHKDPIECA VCLSEFEESE TGRVLPNCQH TFHVDCIDMW
151 FHSHSTCPLC RSLVESLAGI ESTAAARERE VVIAVDSDPV LVIEPSSSSG
201 LTDEPHGSGS SQMLREDSGR KPAAIEVPRR TFSEFEDELT RRDSPASQSF
251 RSPMSRMLSF TRMLSRDRRS ASSPIAGAPP LSPTLSCRIQ MTESDIERGG
301 EESR
```

C

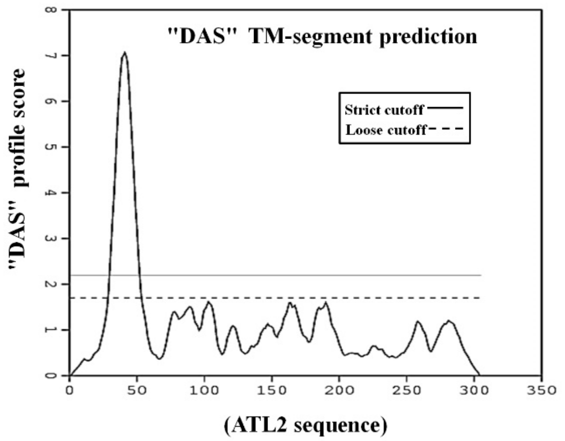

Figure S1. Analysis of ATL2 protein sequence. **(A)** Schematic diagram of ATL2 protein representing membrane domain and cytosolic region. The blue box in N-terminus indicates a transmembrane domain (TM) and RING zinc finger domain (RING) in middle region is represented by the red box. **(B)** Sequence of ATL2. ATL2 consists of 304 amino acids. A transmembrane and RING domain represent by bold character and underline, respectively. **(C)** Analysis of topology of ATL2 protein. Full-length of ATL2 sequence was examined by DAS transmembrane prediction server (https://tmdas.bioinfo.se/). A solid and dashed line indicate strict and loose cutoff for transmembrane prediction, respectively.

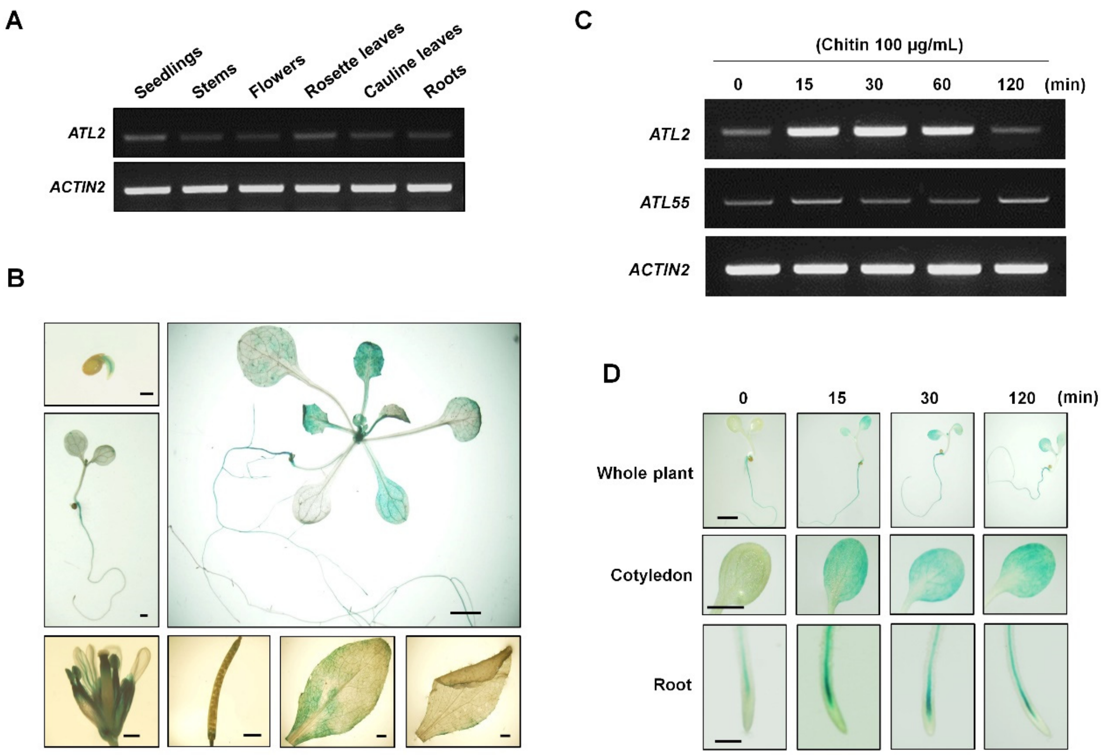

Figure S2. Expression patterns of the *ATL2*. **(A)** Tissue-specific expression of the *ATL2* transcripts. Total RNAs were isolated from 10-day-old seedlings and various tissues, 2-week-old wild-type (roots and rosette leaves), and 5-week-old wild-type plants (stems, flowers, and cauline leaves) grown under long-day growth conditions. *ACTIN2* was used as a loading control. **(B)** Histochemical analysis of *ATL2 promoter-GUS* transgenic plants under normal growth conditions. 2-day-old germinated seed, 7-day-old seedling, 2-week-old plant, flower, silique, rosette leaf and cauline leaf. Scale bars = 1 µm in (2-day-old germinated seed); 2 mm in (7-day-old seedling), (2-week-old plant), (silique), (rosette leaf) and (cauline leaf); 500 µm in (flower). **(C)** The expression patterns of *ATL2* after the addition of 100 µg/mL chitin over the indicated time courses (0, 15, 30, 60, and 120 minutes). Total RNA was isolated from 10-day-old plants harvested at the indicated times after chitin treatment. RT-PCR analysis was performed with *ATL2 and ATL55* gene-specific primers. *ATL55* and *ACTIN2* were used as a negative and a loading control, respectively. **(D)** Histochemical analysis of *ATL2 promoter-GUS* transgenic plants after chitin treatment. 6-day-old seedling plants were treated with chitin (100 µg/mL) for 15, 30, and 120 min before being subjected to histochemical analysis. Photographs were taken of whole plants (top panel), cotyledon (middle panel), and roots (bottom panel) at the indicated times. At least three independent transgenic plants were used for each analysis. Scale bars = 5 mm in the top; 2 mm in the middle; 0.5 mm in the bottom.

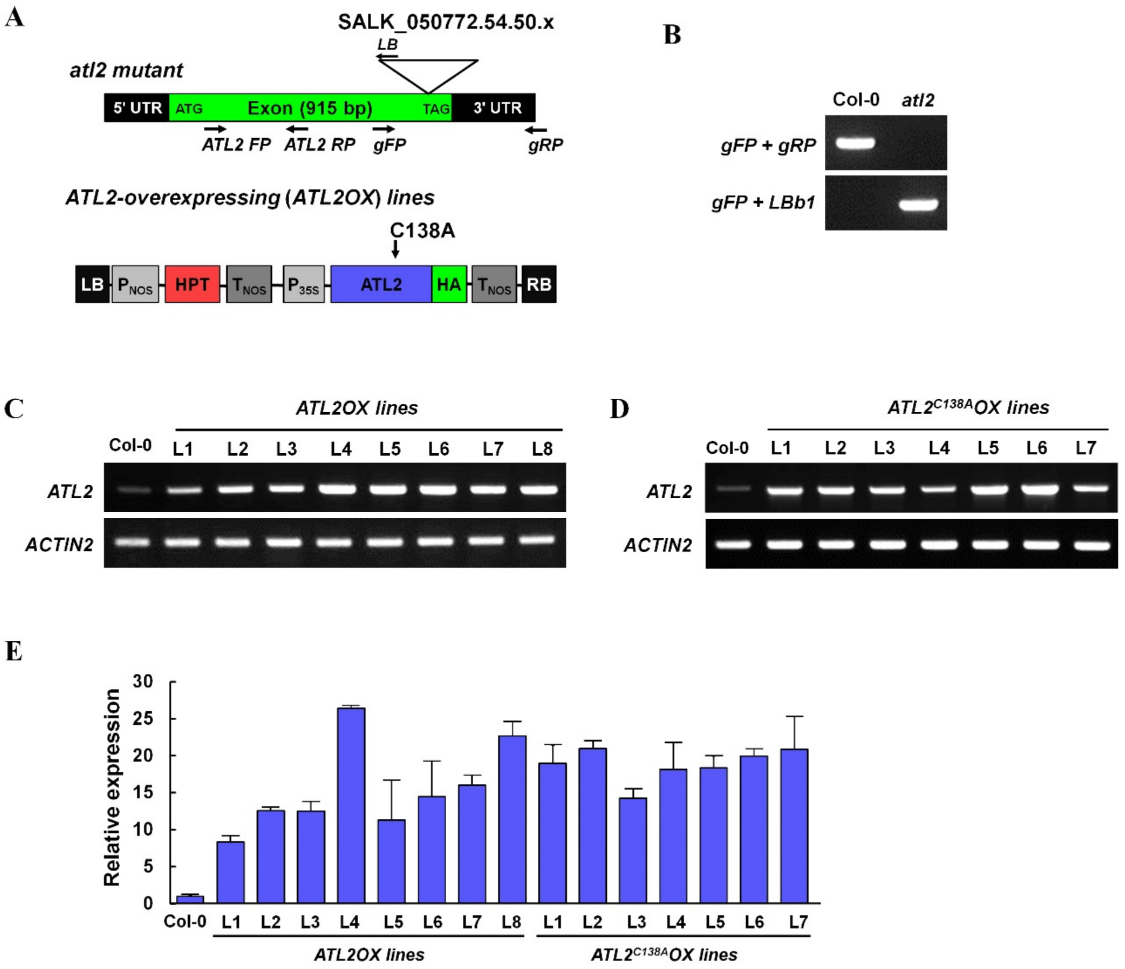

Figure S3. Analysis of homozygous *atl2 mutant, ATL2OX, and* $ATL2^{C138A}OX$ *lines* by PCR reaction. **(A)** Schematic diagram of the T-DNA insertion region (upper, *atl2*) and of the *ATL2*-overexpressing plants driven by *CaMV35S* promoter (lower, *ATL2 OX lines*). *ATL2* consists of one exon. *ATL2 FP* and *ATL2 RP* primers were used for RT-PCR and RT-qPCR analysis. *gFP* and *gRP* were used for genotyping of the *atl2*. Green and black boxes represent exon and untranslated regions (UTRs). An inverted open triangle indicates the position of the T-DNA insertion. The *atl2* (*SALK-050772.54.50.x*) was purchased from the SALK collection. **(B)** Gene-specific (*gFP* and *gRP* primer) and T-DNA-specific (Left border b1; *LBb1*) primers were used (Primers are designated in Figure S3A). Total genomic DNA was isolated from 10-day-old seedling plants. PCR was performed with a set of gene-specific primers (*gFP* + *gRP*; upper) and gene-specific and the T-DNA-specific primer (*gFP* + *LBb1*; lower) for *ATL2* and junction of T-DNA region in *ATL2* gene amplification, respectively. **(C-E)** Isolation of *ATL2*-overexpressing plants. Investigation of *ATL2* transcript levels in *ATL2*-transgenic plants by RT-PCR **(C, D)** and RT-qPCR **(E)** analysis. Total RNA was isolated from 10-day-old plants. *ACTIN2* was used as a loading control and data normalization for RT-PCR and RT-qPCR analysis.

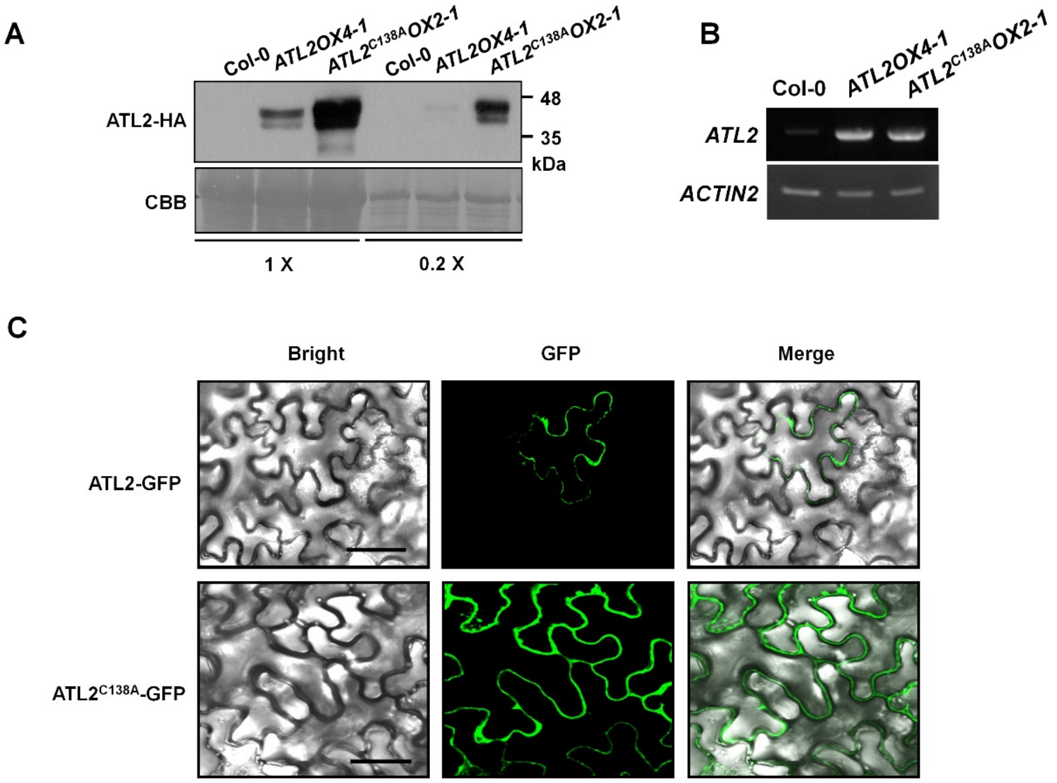

Figure S4. Analysis of E3 ligase activity on ATL2 protein stability. **(A, B)** Comparison of ATL2 stability with mutated ATL2 ($ATL2^{C138A}$) protein (A) and *ATL2* transcript levels between *ATL2OX4-1* and *ATL2^{C138A}OX2-1* (B). Total proteins and RNAs were extracted from each 10-day-old *ATL2OX4-1* and *ATL2^{C138A}OX2-1* overexpressing plants. Western blot analyses were performed with anti-HA antibodies. CBB was used as a loading control. 0.2 X indicates five times dilution from the total protein. RT-PCR was performed with *ATL2*-specific primers. *ACTIN2* was used as a loading control. **(C)** Comparison of ATL2-GFP and $ATL2^{C138A}$-GFP by confocal laser scanning microscopy in *N. benthamiana*. Merge indicates overlapped images of bright and GFP (green) fluorescent signals. *N. benthamiana* leaves were infiltrated with *A. tumefaciens* carrying the indicated DNA constructs, and images were taken by confocal laser scanning microscopy 72 hours post-transfection. Scale bars = 50 μm.

Table S1. The list of primers used in this study.

1. Primer names	2. Primer sequences
3. ATL2 for RT- and qRT-PCR	5. FP (5'-TCGAATGCGCCGTTTGTTTATCGG-3')
4. (ATL2 FP/ATL2 RP)	*RP (5'-ATTCAATCCCGGCGAGAGACTCAA-3')*
ATL55 for RT- and qRT-PCR	*FP (5'-GTCGTCGGCTCAATCATACTC-3')*
	RP (5'-CCTCTGTTTCGTCCTCATCTT C-3')
ATL2 for genotyping (gFP/gRP)	*FP (5'-ACTTGGTCCACTAACGCGTAG-3')*
	RP (5'-AGTTGTGATTGCGGTTGATTC-3')
ATL2 for HA-tagged overexpression	*FP (5'-GTACCATGGTAATGAACTCCAACGACC-3')*
	RP (5'-GTAAGATCTTTCCTACTCTCTTCTCCTCCCCC-3')
ATL2 Promoter for GUS staining	*FP (5'-AAAAAGCAGGCTCAAAGGAATGAAGCC-3')*
	RP (5'-AGAAAGCTGGGTACGGATCCTGGTCG-3')
ATL2 for MBP fused protein expression	*FP (5'-GTAGTCGACATGAACTCCAACGACCAGG-3')*
	RP(5'-GTACTGCAGTCACCTACTCTCTTCTCCTCCCCG-3')
ATL2 for GFP construct	*FP (5'-AAAAAGCAGGCTCAATGAACTCCAAC-3')*
	RP (5'-AGAAAGCTGGGTACCTACTCTCTT-3')
Left Border b1	*(5'-GCGTGGACCGCTTGCTGCAACT-3')*
ACTIN2	*FP (5'-ACGAGCAGGAGATGGAAACCTCAA-3')*
	RP (5'-TCTTCATGCTGCTTGGTGCAAGTG-3')
A. brassicicola CutA	*FP (5'-CACTGCGCCCAATGATGAAC-3')*
	RP (5'-GTAGCCGAACAACACGACACC-3')
